# Robust Traffic Light and Arrow Detection Using Digital Map with Spatial Prior Information for Automated Driving

**DOI:** 10.3390/s20041181

**Published:** 2020-02-21

**Authors:** Keisuke Yoneda, Akisuke Kuramoto, Naoki Suganuma, Toru Asaka, Mohammad Aldibaja, Ryo Yanase

**Affiliations:** 1 Institute for Frontier Science Initiative, Kanazawa University, Kanazawa, Ishikawa 920-1192, Japan; 2 Department of Mechanical Systems Engineering, Tokyo Metropolitan University, Hino, Tokyo 191-0065, Japan

**Keywords:** image processing, traffic light detection, intelligent transportation system

## Abstract

Traffic light recognition is an indispensable elemental technology for automated driving in urban areas. In this study, we propose an algorithm that recognizes traffic lights and arrow lights by image processing using the digital map and precise vehicle pose which is estimated by a localization module. The use of a digital map allows the determination of a region-of-interest in an image to reduce the computational cost and false detection. In addition, this study develops an algorithm to recognize arrow lights using relative positions of traffic lights, and the arrow light is used as prior spatial information. This allows for the recognition of distant arrow lights that are difficult for humans to see clearly. Experiments were conducted to evaluate the recognition performance of the proposed method and to verify if it matches the performance required for automated driving. Quantitative evaluations indicate that the proposed method achieved 91.8% and 56.7% of the average f-value for traffic lights and arrow lights, respectively. It was confirmed that the arrow-light detection could recognize small arrow objects even if their size was smaller than 10 pixels. The verification experiments indicate that the performance of the proposed method meets the necessary requirements for smooth acceleration or deceleration at intersections in automated driving.

## 1. Introduction

Automated vehicle technologies are considered to be the next generation transportation system. Many companies and research organizations are involved in the research and development of such technologies. Recent automated vehicle technologies focus more on urban driving, which is a mixed transportation environment with human drivers. Public road demonstration experiments have been carried out in the U.S., European, and Asian countries since the mid-2000s [1,2,3]. Automated driving in mixed environments human driven vehicles, pedestrians, and cyclists requires recognition of surrounding objects autonomously and decision making according to traffic rules. The following sensors are mainly mounted on automated vehicles for surrounding recognition.

- Ranging sensors: light detection and ranging (LiDAR), millimeter-wave radar (MWR)
- Imaging sensor: camera
- Positioning sensor: global navigation satellite system and inertial navigation system (GNSS/INS).

The above mentioned sensors make it possible to observe surrounding transportation environments. In addition, recent automated driving technologies rely on high-definition maps (HD maps) which include the precise position of static road features such as lane boundaries, lane centerlines, traffic signs, and traffic lights. By referring to the predefined road features, it is possible to reduce false surrounding recognitions and implement accurate decision making, considering road structures. The following functions must be implemented in order to achieve automated driving on public roads.

1. Self-localization: Estimating the position of the vehicle on the HD map in the accuracy of decimeter-level
2. Surrounding perception: Recognizing static/dynamic objects including traffic participants and road objects (e.g., lane marking, traffic signals)
3. Motion planning: Designing the optimal collision-free trajectories following the traffic rules
4. Motion control: Determining adequate control signals such as steering, acceleration and braking.

This study focuses on traffic light (TL) detection using HD maps. There are many studies on TL detection by using vision-sensors in intelligent transportation system. TL is one of the most important road features in order to decide an approaching the intersection. Although HD maps have the information of TL positions, the vehicle must recognize the current state of TLs in real time because it changes dynamically. For safety deceleration, it is necessary to recognize the current state of TLs at distances over 100 m. The required recognition distance can be estimated by calculating the braking distance from the vehicle to the stop line after smoothly recognizing the TL state. In studies on vehicle control [4,5], the deceleration without discomfort to passengers is approximately 0.1 G ( =0.098 m/s${}^{2}$). For example, when the vehicle decelerates by 0.1 G while traveling at a velocity of 50 km/h, the braking distance is approximately 98 m. Furthermore, the recognition distance may increase further when considering the case where the TL is located at a position away from the stop line. Recognizing TLs in the ranges exceeding 100 m is required to make a natural intersection approach in automated driving. In order to implement a practical method of TL recognition, it is necessary to discuss the effectiveness of the methods, considering the trade-off between the required performance and the hardware specification. For example, installing a high resolution camera or a telephoto lens is an easy solution to increase the recognition distance. However, increasing the resolution may increase the processing time. In addition, the field of view is narrowed and TLs may be left out. From the point of view of implementing a recognition method, it is important how to recognize small pixel objects.

On the other hand, in automated driving using HD maps, the self-localization module precisely estimates the vehicle pose by map-matching using a range sensor or image sensor [6,7,8]. Generally, position accuracy of approximately 0.1 to 0.2 m is considered to be necessary for decision-making and path planning in automated driving. Assuming that the precise vehicle position on the digital map is estimated, a region-of-interest (ROI) location for the TLs can be calculated using the registered TL position and current vehicle pose. Extracting the ROI makes it possible to reduce the search region of TLs. It is then possible to reduce false detections such as false-positive and false-negative detections, and computational costs [9,10,11,12]. In addition to improving recognition performance, associating TLs registered on a map with TLs in an image is an important aspect of the map-based recognition. In the decision making using the HD maps, it is necessary to grasp the state of the relevant TLs, in order to make an approach decision at the intersection.

The purpose of this study is to achieve small object recognition for both TLs and arrow TLs. Different types of candidate detectors are introduced to realize robust lighting area detection. In addition, in order to implement robust arrow recognition, the proposed method uses spatial information of the positional relationship of lights in TLs as prior information obtained from the HD map. The proposed method is specialized in the recognition of small-sized objects, and evaluate recognition performance for actual driving data. Moreover, verification experiments for automated driving were carried out by introducing the proposed method to investigate the validity of the method.

The rest of this paper is composed as follows. Section 2 describes related works. Section 3 introduces the proposed TL detection method. Section 4 presents evaluations for the proposed method with some discussions. Finally, Section 5 concludes the paper with some remarks.

## 2. Related Works

Table 1 summarizes the reported results of major research cases on the TL recognition. Based on the state-of-the-art researches, the recognition procedure can be described as follows:

1. Determine the search region: A region-of-interest (ROI) is extracted from the captured image by using the predefined map.
2. Extract candidate objects: Circular lighting areas or rectangular objects are extracted from the search region as candidate TLs.
3. Classify the state of the candidates: Simple color filtering or machine learning algorithms identify lighting colors and arrow light directions.

Although different approaches have been developed, most of the methods involve extracting candidates according to their specific color spaces [14,20] and circular shape [10,21], and identifying them as TLs or arrow TLs. Regardless of the country, the TLs mainly consists of circle and arrow shaped lights. In the case of the ROI-based recognition, detection of circular objects is one of the effective approaches to recognize lighting areas because the search region is limited in it. According to Table 1, many methods adopted a blob detector which extracted candidate objects by binarizing the image and segmenting pixels [11,13,14]. It can detect circular objects even if their size is a few pixels. Then, the recognition of the whole shape of the TLs are implemented using specific shape matching and machine learning. Moreover, the effect of introducing object tracking to stabilize the recognition result has been reported [14,22]. In recent years, there have been reports of cases in which performance is improved upon using deep neural network (DNN) [11,14,16,18,23]. In order to detect arrow TLs, machine learning-based detector is a key solution. As shown in Table 1, it has been reported that these methods can recognize TLs at distances exceeding 100 m, with a recognition rate of approximately 90%. However, it is difficult to directly compare each performance, because the specifications of the camera (image sensor, resolution, and field of view), the driving scene, and the quality of data are different. Herein, we discuss algorithm limitations by comparing the pixel size of recognizable objects.

On the other hand, assuming that our algorithm will be introduced into the automated vehicles, real-time proceeding is important for decision making. In addition, in order to reduce the delay in recognition, it is necessary to recognize TLs in an appropriate processing time in accordance with the velocity of the vehicle. For example, when traveling at a velocity of 50 km/h, a vehicle moves about 14 m per second. Then, it is important to estimate the required time in consideration of the responsive deceleration for practical development.

In our previous study [24], the TL recognition method was proposed using sped up robust features (SURF) [25]-based circular object detector. It can detect circular objects like a blob detector without binarization, therefore the robust candidate extraction is expected. In addition, the proposed method estimates the existence probability of lighting objects in an image. It has the advantage of reducing false positive detections which caused by surrounding lighting objects. In this work, we improve our method by integrate the typical candidate detectors such as a circular detector using SURF, a blob detector, and the TL shape detector. In particular, we investigate the performance and limitation of the arrow detection method by introducing prior information in order to robustly detect arrow TLs. Moreover, we verify that the performance requirements for the recognition distance are satisfied, by performing automated driving on an urban road. The followings are the contributions of this paper.

- The performance evaluation is performed in challenging dataset including small objects of TLs and arrow TLs in the day and night.
- The effectiveness of prior information is evaluated with respect to the performance in recognition of distant TLs.
- Verification results are presented by introducing the proposed method in automated driving.

## 3. Proposed Traffic Light Detection

### 3.1. Traffic Light Detection

**Figure 1:**
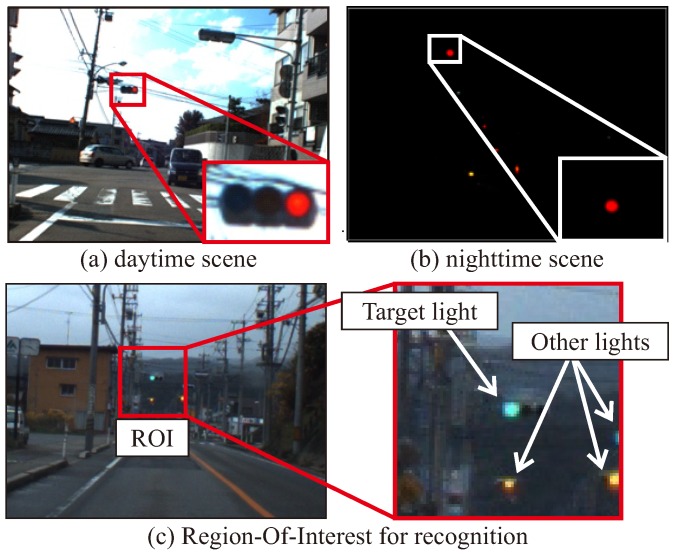
Typical traffic light image at different brightness and region of interest (ROI) image.

Before describing the proposed algorithm, the problem of the TL recognition addressed in this study is explained. In the TL recognition, the task is to recognize the state of the TLs in the image that corresponds to the HD map. As shown in Figure 1a,b, it is necessary to properly recognize the lighting status of TLs both in the day and the night. On the HD map, the position information of the TLs is recorded individually, and then the TL positions in the camera image can be calculated from the position of the TL and the vehicle. Figure 1c indicates the typical ROI image which is extracted by the coordinate transform using the HD map for a driving image. As in the enlarged image in Figure 1c, the extracted ROI image may include TLs other than the ones, and background lighting objects. In implementing automated driving at intersections, the purpose is to recognize the TL associated with the ROI. Therefore, if a different TL is recognized in the specific ROI, it will be a false-positive detection.

**Figure 2:**
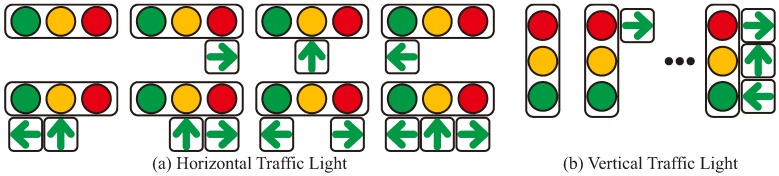
Traffic light patterns that can be recognized in the proposed method.

Figure 2 shows the TL patterns to be recognized by the proposed method. This study focuses on the recognizing TLs in the Japanese traffic environment. We deal with the TL patterns that include three basic types of lights (green, yellow, red) and three types of arrow lights (left, straight, and right) that exist depending on the road environment in Japan. In addition, because there are horizontal and vertical TLs depending on the area in Japan, the proposed method recognizes these patterns as well. As a special case, there are cases where arrow lights in different positions and arrow lights in different directions as shown in Figure 2, are installed in the actual environment. Evaluation of recognition performance for such special situations has not been performed in this work, but it can be easily extended by using the digital map information described herein, as a prior information. Although the proposed method will be evaluated for Japanese traffic images in this work, the proposed method is able to apply to general traffic lights which consist of circular lights and arrow lights. In the proposed method, the recognition distance can be improved if the arrangement pattern of the signal light and the arrow light is known for the target TLs as shown in Figure 2.

### 3.2. Predefined Digital Map

A highly-precise digital map is maintained by a growing number of professional mapping companies. Accurate positioning systems, combined with cameras and LiDAR sensors can be possible to generate a precise 3-D maps which contains latitude, longitude and altitude reflectivity. For the purpose of the TL detection, location data of each TL was recorded into the map information. The method described in this paper uses the following information as prior map information:

- the 2-D TL positions (latitude, longitude, and heading direction)
- attribute information of the TL directions (horizontal or vertical)
- attribute information of the type of the TL patterns (see Figure 2a).

Although the exact altitude of the TLs is not used as a prior information, they are installed at a height of approximately 5.0 m above the ground surface on the Japanese road environment. Therefore, the recognition process is performed by considering a height of 5.0 m as a reference height, and providing a margin that assumes a road gradient. Although the standard height of TLs is specified in Japan, the standard height should be different in other countries. It is necessary to set an appropriate height according to the target country or to set a wider recognition area in the image when the height information is unknown.

### 3.3. Method

**Figure 3:**
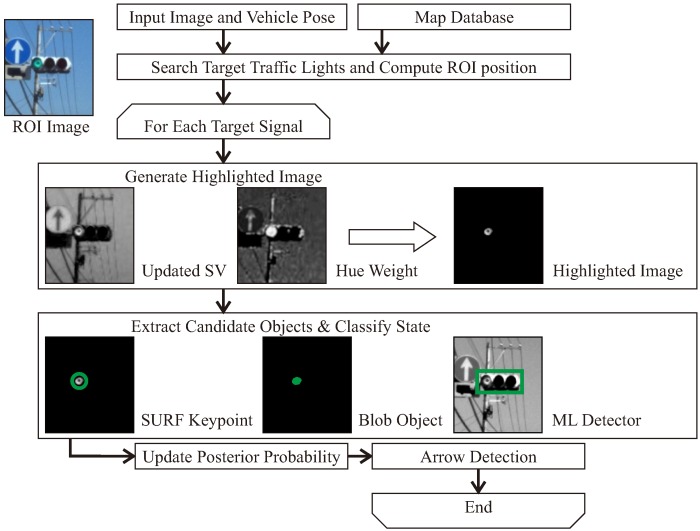
Flowchart of the proposed method.

Figure 3 illustrates a flowchart of the proposed method. It mainly consists of the following five procedures:

1. Search target TLs and compute ROI
2. Generate a highlighted image as a feature image which emphasizes the lights of TLs in the ROI image
3. Extract candidates for TL lights in the generated highlighted image using three types of different methods
4. Compute the probability of existence area containing TLs using a time-series processing
5. Recognize the arrow light, if the target TL has attribute information of arrow light.

**Figure 4:**
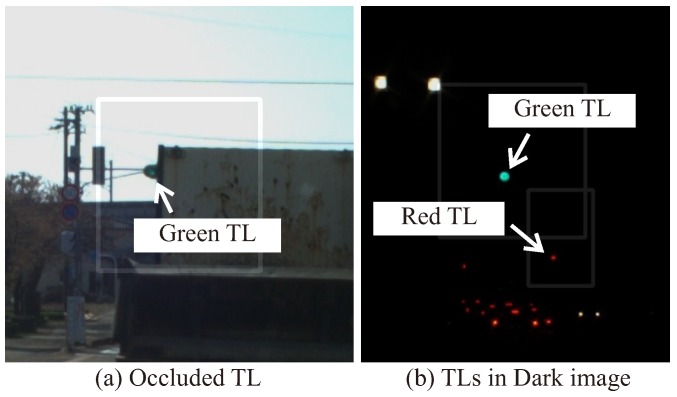
Typical traffic light (TL) images in occluded and dark scenes.

As described in Section 2, most of the existing recognition methods mainly use individual features such as circular objects, blob regions, and overall shapes using a machine learning (ML) detector to recognize the TL candidates. The proposed method combines them to detect candidates for robust implementation. By using detection methods that focuses on the lighting area of the TLs, it is possible to recognize them even if the overall shape of the TL is not visible, such as during occlusion or at night time. Figure 4 shows typical driving images in an occluded and a dark scenes. Occlusion of TLs is caused by surrounding other vehicles such as a preceding vehicle, a bus, and a tuck. As shown in Figure 4a, there is a situation that the occluded situation where it is difficult to see the whole shape of the TL. However, it is necessary to recognize the TL state only from the lighting area. The situation where such an overall shape cannot be visually recognized is the same even at night as shown in Figure 4b. Section 4 evaluates the contribution of each method by comparing the recognition performances.

Arrow detection requires recognition of directions, which is affected by unclear images. In order to improve such distant arrow recognition, we propose an arrow recognition method using prior information of the HD map. In addition, this study verifies the effects of using prior information in the arrow light recognition. The algorithms are described in detail in the following section.

### 3.4. Coordinate System and Traffic Light Selection

**Figure 5:**
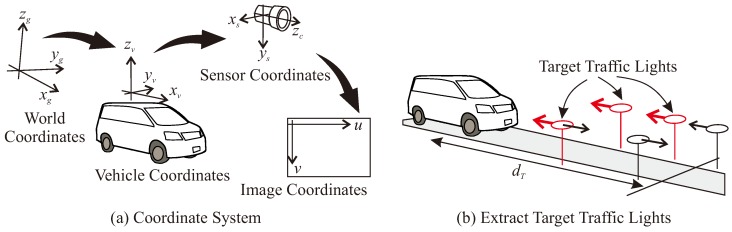
Coordinate systems and traffic light selection.

Figure 5 illustrates the coordinate systems considered in this work. The latitude and longitude values are converted to 2-D $x_{g}-y_{g}$ space in the universal transverse mercator (UTM) coordinate system. The world coordinate system is defined as $x_{g}$, $y_{g}$ and $z_{g}(=$altitude). The vehicle coordinate system is centered at the rear wheels. The front direction is $x_{v}$, the left direction is $y_{v}$ and the upper direction is $z_{v}$. In a similar way, sensor coordinates ($x_{s}-y_{s}-z_{s}$) and image coordinates (u-v) are defined as shown in Figure 5a. The world, vehicle and sensor coordinates can be transformed by using rotation and translation matrices. Sensor and image coordinates are transformed by using intrinsic parameters.

Among the TLs that appear in the frontal camera image, the TLs that are within a certain distance $d_{T}$, and whose heading angle difference is within a certain degree $\theta_{T}$, are extracted as target TLs to be recognized. The target TLs are extracted based on the distance parameter $d_{T}$ m from faced traffic signals as shown in Figure 5b. In Figure 5b, the red TLs are the extracted target TLs.

### 3.5. ROI Clipping

The ROI image is clipped for each target TL. The location of the ROI can be calculated based on the current pose and the map database. A global TL position $x_{w}=\left[x_{w},y_{w},z_{w},1\right]$ is converted to $x_{v}=\left[x_{v},y_{v},z_{v},1\right]$ and $x_{s}=\left[x_{s},y_{s},z_{s},1\right]$ by the vehicle pose and extrinsic parameters of the camera.

(1) $$\begin{array}{cc} x_{v} & =R_{wv}x_{w} \end{array}$$

(2) $$\begin{array}{cc} x_{s} & =R_{vs}x_{v}, \end{array}$$

where $R_{wv}$ and $R_{vs}$ are 4×4 homogeneous transformation matrices for converting world-to-vehicle coordinates and vehicle-to-sensor coordinates, respectively. As described above, if there is no information on the absolute height of the TL, the general TL height is used to compute $z_{v}$, assuming a flat surface. In this case, the height $z_{v}$ is calculated by the following equation:

(3) $$z_{v}=z_{0}-x_{v}\tan\phi ,$$

where $z_{0}$ is the general TL height from the road surface (e.g., $z_{0}=5.0$m), and ϕ is the pitch angle of the vehicle. A pixel position u,v of the signal is then calculated based on the intrinsic parameters and the following set of equations:

(4) $$\begin{array}{cc} x^{\prime \prime } & =x_{s}/z_{s},\;y^{\prime \prime }=y_{s}/z_{s},\;r^{2}=x^{\prime 2}+y^{\prime 2} \end{array}$$

(5) $$\begin{array}{cc} x^{\prime \prime } & =x^{\prime }\left(1+k_{1}r^{2}+k_{2}r^{4}\right)+2p_{1}x^{\prime }y^{\prime }+p_{2}\left(r^{2}+2x^{\prime 2}\right) \end{array}$$

(6) $$\begin{array}{cc} y^{\prime \prime } & =y^{\prime }\left(1+k_{1}r^{2}+k_{2}r^{4}\right)+p_{1}\left(r^{2}+2y^{\prime 2}\right)+2p_{2}x^{\prime }y^{\prime } \end{array}$$

(7) $$\begin{array}{cc} u & =f_{x}x^{\prime \prime }+c_{x},\;v=f_{y}y^{\prime \prime }+c_{y}, \end{array}$$

where $f_{x},f_{y},c_{x},c_{y},k_{1},k_{2}$ are intrinsic parameters of the camera. The ROI is defined as a rectangle with a width $w_{roi}$, and a height $h_{roi}$ centered at the pixel u,v.

(8) $$\begin{array}{cc} w_{roi} & =k_{roi}\frac{f_{x}s_{s}}{z_{s}} \end{array}$$

(9) $$\begin{array}{cc} h_{roi} & =k_{roi}\frac{f_{y}s_{s}}{z_{s}}, \end{array}$$

where $s_{s}$ is the size of a TL and $k_{roi}$ is a constant parameter to determine a scale of the ROI.

### 3.6. Highlighted Image Generation

**Figure 6:**
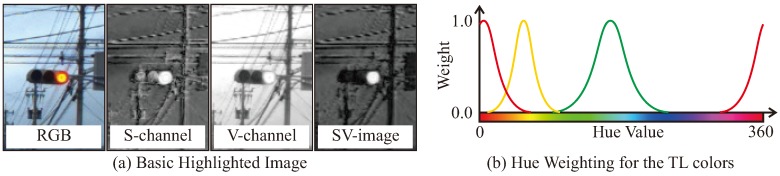
Highlighted image generation.

The lighting areas of the TLs have higher brightness and saturation compared to other objects. Therefore, the highlighted image can be generated by multiplying the saturation image by the brightness image. In order to extract lighting areas, RGB images are converted into HSVcolor space. The lighting area of the TL gets highlighted as shown in Figure 6a. The highlighted images have higher brightness and saturation to emphasize the lighting areas of TLs. However, in some cases, the highlighted image cannot emphasize the lighting area sufficiently, especially for the lamp-type TLs. In addition, in recognition of distant TLs where the image is unclear, there is a possibility that false-positive detections may occur under the influence of background noise. In order to solve these problems, we have previously reported a method that can reduce false-detection, such as false-positive and false-negative, in distant places, by correcting and weighting the highlighted images [26]. The following processes are suggested to emphasize TLs:

- Normalize the brightness value to emphasize the lighting.
- Update the saturation value to eliminate background noise.
- Weighting with respect to hue value, close to the lighting color of traffic signals.

The first operation updates the image brightness. The brightness value is normalized using the following equation:

(10) $$V_{m}\left(u,v\right)=k_{v}\bar{V}+\frac{\sigma_{v}}{\sigma }\left(V\left(u,v\right)-\bar{V}\right),$$

where V(u,v) and $V_{m}\left(u,v\right)$ are the original brightness from the HSV image and modified brightness value at pixel (u,v), respectively. $\bar{V}$ is the average brightness of the original brightness image V. σ is the standard deviation for V, and $\sigma_{v}$ is the modified standard deviation for the updated image $V_{m}$. $k_{v}$ is a constant parameter that increases the average brightness.

The second operation updates the saturation values. The lighting area of the traffic signal generally has saturation values above a certain value. The pixels with saturations lower than this value are reduced using the following equation:

(11) $$S_{m}\left(u,v\right)=S\left(u,v\right)\cdot \frac{1}{(1+\exp\left(-a_{s}\left(S\left(u,v\right)-b_{s}\right)\right)},$$

where S(u,v) and $S_{m}\left(u,v\right)$ are the original saturation values from the HSV and modified saturation value, respectively. $a_{s}$ and $b_{s}$ are constant parameters of the sigmoid function to reduce the saturation value. $S_{m}$ and $V_{m}$ are used to generate the highlighted image instead of the SV-image.

The third operation multiplies the pixel values of the highlight image with weight, with respect to the hue values. Figure 6b shows the definition of weighting value. It means that the hue value closest to the lighting color of the traffic signal has a higher weight value.

(12) $$\begin{array}{cc} W_{G}\left(u,v\right) & =\exp\frac{-{\left(H\left(u,v\right)-\mu_{G}\right)}^{2}}{2\sigma_{G}^{2}} \end{array}$$

(13) $$\begin{array}{cc} W_{Y}\left(u,v\right) & =\exp\frac{-{\left(H\left(u,v\right)-\mu_{Y}\right)}^{2}}{2\sigma_{Y}^{2}} \end{array}$$

(14) $$\begin{array}{cc} W_{R}\left(u,v\right) & =\exp\frac{-{\left(H\left(u,v\right)-\mu_{R}\right)}^{2}}{2\sigma_{R}^{2}} \end{array}$$

(15) $$\begin{array}{cc} H_{w}\left(u,v\right) & =\max(W_{G}\left(u,v\right),W_{Y}\left(u,v\right),W_{R}\left(u,v\right)), \end{array}$$

where H(u,v) is the original hue value from the HSV. $W_{*}\left(u,v\right)$ is the obtained weight value, $\mu_{*}$ is a mean value and $\sigma_{*}$ is the standard deviation for weighting for the corresponding colors. $\mu_{*}$ and $\sigma_{*}$ should be determined according to the color balance of the camera.

### 3.7. Candidate Extraction and State Classification

After the generation of the highlighted image, a lighting area detection is applied to the obtained image. As mentioned in Figure 3, the proposed method introduces three types of methods to extract candidate lighting objects.

**Figure 7:**
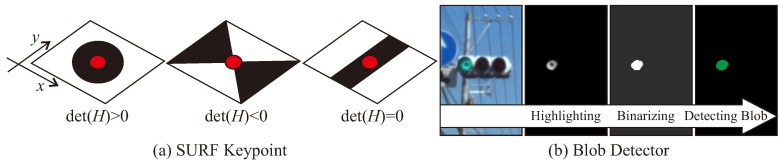
Candidate extraction.

The first method is the circle detector. The shape of the lighting area is generally circular shape in the image. A method based on the Hough transform has been adopted to extract circular candidates [21]. However, because a clear circular area cannot be obtained in the image for a distant TL, a blob detector described later was adopted in many works. SURF keypoints have a Hessian matrix *H*, the types of the edges can be categorized by using det(H) as shown in Figure 7a. Candidate circle areas can be extracted as keypoints with det(H) higher than the threshold value $H_{min}$. This approach can extract circular objects robustly, because SURF is a robust keypoint for illumination change and scale change.

The second method is the blob detector. In the feature image, areas with higher pixel values are distributed near the lighting areas. These areas can be extracted by binarizing and segmenting the image as shown in Figure 7b. Such a method of extracting candidate objects by binarization according to the brightness, saturation and color characteristics has also adopted in many related works [11,13,14]. Results are expected to be similar to circle detection, but the blob detector is expected to be better than the circle detector, when a part of the lighting part is saturated and cannot be visually recognized as a circle shape in the feature image. However, the blob detector is sensitive to threshold adjustment for binarization. If there is a signboard with a color close to the lighting color in the background, it may be detected as false postives. In addition, when the brightness of the lighting area in the image changes due to the influence of the surrounding light, it may be false negatives.

The third method is the ML-detector. Because the detection is performed using camera images, the brightness of the whole image may be affected by the influence of the surroundings, such as sunlight and environmental light. In such cases, the lighting area of the TLs cannot be sufficiently emphasized in the highlighted image. In order to recognize such TLs with lower brightness, it is effective to focus on the whole shape of the TLs together. Generally, machine learning is a common approach to detect such shape of the TL. In the proposed method, the OpenCV cascade detector trained by AdaBoost [27] is used as one of the CPU-based detectors for the TL recognition. In recent years, DNN-based detectors have shown high recognition performance in general object recognition [17,28]. DNN models such as SSD [17] and YOLOv3 [28] are known as typical networks for detecting objects in real-time. However, because the DNN model requires GPU processing, it is necessary to select an appropriate ML-Detector in consideration of the trade-off between computational resources and recognition performance.

The lighting states are classified in the detected objects by these methods, and determined as final candidates. In order to eliminate false-positive detections, objects with comparatively smaller and larger radius are deleted based on the pre-calculated radius $r_{l}=0.5f_{x}s_{l}/z_{s}$ from the HD map. Here, $s_{l}$ is the diameter of the lamp of the TL. The accepted candidates are extracted according to the minimum radius $k_{min}r_{l}$, and the maximum radius $k_{max}w_{l}$, based on the parameters $k_{min}$ and $k_{max}$. In addition, in order to reduce the processing time, the ML-detector is used only when the circle and blob detectors have not detected any objects.

The lighting color of the candidate object needs to be classified from the hue and brightness distribution of the lighting area. In the proposed method, histograms of the highlighted image and hue image are created for the detected lighting area, and then the AdaBoost classifier is trained using the normalized histograms at the maximum frequency as a feature vector. The generated classifier recognizes the lighting state via four classes, namely Green, Yellow, Red, and Background.

### 3.8. Probability Updating

**Figure 8:**
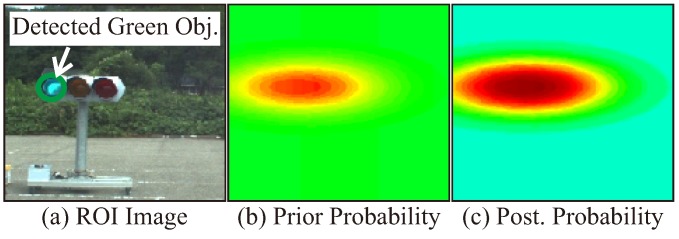
Probability updating.

The candidates detected in the ROI are potential objects of the target TL. In order to output a likely object from the obtained candidates, a time-series tracking process is performed by computing existence probability. In [14], multi-object tracking is implemented to improve recognition accuracy. In the proposed method, the whole shape of the TL is not always recognized. Therefore, the probability is estimated by calculating the existence probability of the object in the 2-D image space, instead of general tracking using the target as a mass point. The existence probability is computed using a binary Bayes filter (BBF). The following equation shows a relationship between the log-odds *l* and the probability *p* for the *i*-th target signal at time *t*.

(16) $$p_{i}\left(u,v|z_{1:t},x_{1:t}\right)=\frac{1}{1+\exp(-l_{t,i}\left(u,v\right))},$$

where $z_{1:t}$ and $x_{1:t}$ are the observation and the vehicle state until time *t*, respectively. (u,v) is the pixel location in the image and $l_{t,i}\left(u,v\right)$ is a log-odds value at the pixel (u,v) for the *i*-th TL. The log-odds can be updated by additional computation in BBF.

(17) $$l_{t,i}^{Prior}=\alpha \;l_{t-1,i}^{Post}+l_{t,i}^{Obs},$$

where $l_{t-1,i}^{Post}$ is the posterior log-odds at the previous time for the *i*-th target signal. The initial value of $l_{t,i}^{Prior}$ is set to 0. α is the decay rate for the previous posterior probability. $l_{t,i}^{Obs}$ is calculated based on the position and size of the obtained candidates. Figure 8 shows a typical example of the probability updating. For each detected candidate, the rectangular area where the TL may exist is calculated, and the observation distribution $l_{t,i}^{Obs}$ is determined based on the Gaussian distribution around the rectangular areas. Then, it is possible to estimate a likely region by performing time-series processing.

### 3.9. Arrow Signal Recognition

**Figure 9:**
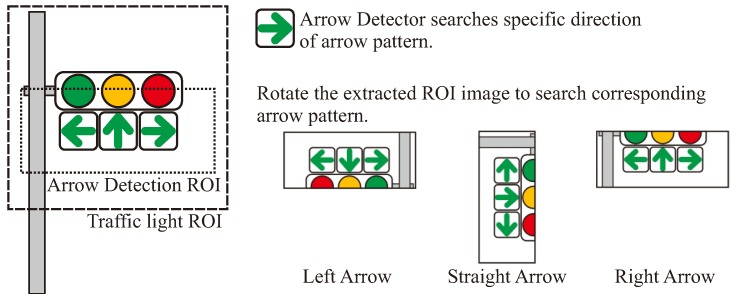
Arrow light recognition.

In addition to the TL detection, an arrow signal is recognized when the TL has the attribute of arrow lights in the HD map. In Japanese traffic environment, arrow lights are generally lit at red and yellow TLs. After detecting a yellow or red signal, an arrow detection ROI is determined as shown in Figure 9. In the recognition process, the right-arrow detector is trained in advance using AdaBoost (cascade detector in OpenCV), and then it is applied to the extracted ROI. In order to detect left/straight arrows, the ROI image is rotated and the same detector is used to search objects.

### 3.10. Prior Information using Digital Map

By using the proposed method described above, the TL recognition is realized by detecting the candidate objects, classifying the lighting color, and computing the confidence using the existence probability. This work further improves the recognition performance, especially for distant arrow lights, by utilizing the prior information given in the digital map.

In the TL recognition, when there are multiple candidate objects, it is possible to weight candidates according to the distance of the TLs in the probability updating procedure. It is expected to reduce false-positive detections in background.

**Figure 10:**
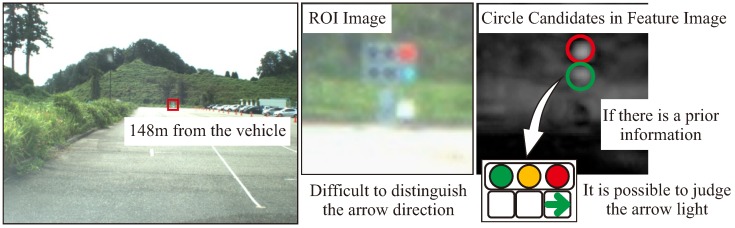
Arrow light recognition using prior information.

On the other hand, in arrow recognition, recognition can be improved by providing the pattern of the target TL from Figure 2 as prior information. For example, Figure 10 illustrates the typical arrow recognition scene. In the recognition of a distant arrow light, if it is difficult to visually recognize the direction of the lighting arrow, it may cause false-positives or false-negatives. In Figure 10, it can be seen that some arrow lights are lit in the ROI image, but it is difficult to distinguish the directions. In this case, because the lighting parts of the arrow light is crushed, a candidate point may be detected at the arrow TL as well as the candidate TL. Normally, this detected candidate can be a false-positive detection of a green signal. However, if information on the relative positional relationship of the arrow lights at the TL is provided as a prior information, it is possible to distinguish the direction of the arrow lights. Our work evaluates how this prior information contributes to the recognition of TLs and arrow lights by the proposed method.

## 4. Evaluations

### 4.1. Condition

**Figure 11:**
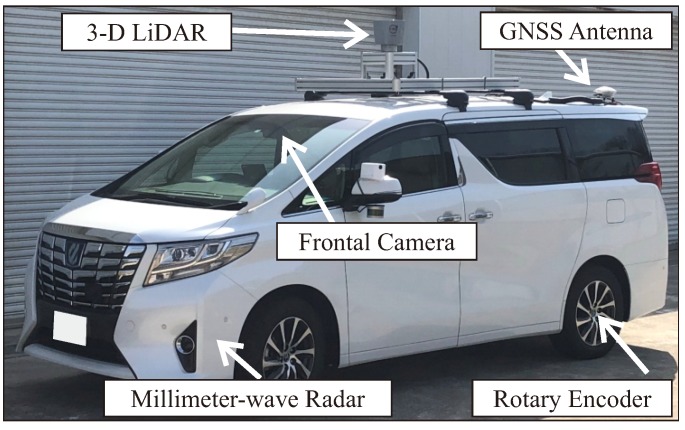
Experimental vehicle.

Experiments have been carried out to evaluate the effectiveness of the proposed method with actual driving data. Some driving data have been collected using the automated vehicle owned by our group. Figure 11 shows our automated vehicle. This automated vehicle was equipped with some sensors such as LiDAR, MWR, GNSS/INS and camera to observe its surroundings. A 3-D LiDAR Velodyne HDL-64E S2 with 64 separate beams was mounted on the vehicle to take measurements of the environment. It measured the 3-D omnidirectional distance at a frequency of 10 Hz. An Applanix POS/LV220 coupled GNSS and INS was mounted on the vehicle. It provided an accurate position (latitude, longitude and altitude) and orientation (pitch, yaw, roll) at 100 Hz. In addition, in order to observe the traffic lights, the vehicle was equipped with a mono-camera Pointgrey Flea2, which provided a 1280 × 960 pixel resolution at 7.5 Hz. There were lamp type and LED type TLs to be recognized. Because the LED type TLs blink at high speeds, the TL may have been turned off when shooting with the camera, depending on the shutter speed. To avoid this problem, the shutter speed was limited from 10 ms to 20 ms for the auto-exposure function. As a result, the image of a dark scene such as the evening was almost dark as shown in Figure 1b and the shape of the traffic light could not be seen.

This vehicle had various functions necessary to enable automated driving in an urban area, and has actually been running in Japan for several years. In previous works, real-time localization algorithms have been developed using different types of sensors such as 3-D LiDARs, cameras or MWRs [7,29,30]. Therefore, it is assumed that the precise vehicle pose has been estimated using such localization algorithms in this evaluation.

**Figure 12:**
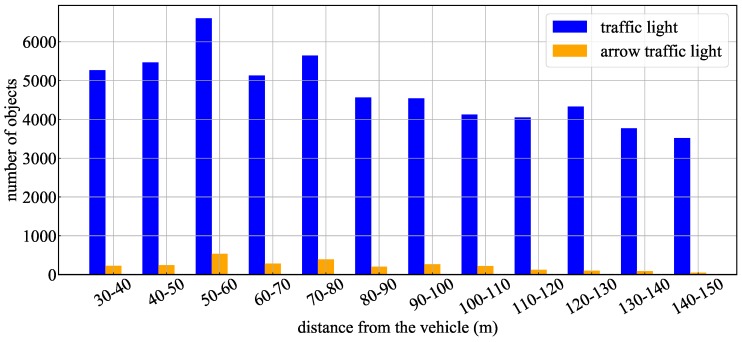
Histogram of number of test data in different distances from the TL.

Table 2 shows the number of images in the train and test dataset. These datasets were recorded at Kanazawa-city and Suzu-city in Ishikawa, Japan. As described in Section 3.1, the TL recognition aims to recognize a total of six states, three states of TLs (green, yellow, and red) and three states of arrow TLs (left, straight, and right). The training data were used to train the overall shape of the traffic light and the arrow light detector using machine learning. These data consisted of images measured during the daytime as visible data of the overall shape of the TL. On the other hand, the test dataset consisted of not only the daytime scene, but also the dark scene which was images measured in the early morning and evening hours. In addition, Figure 12 shows the frequency distribution of test data for distances from 30 m to 150 m. Although the ratio of the arrow TL data was small overall, the test data were distributed almost uniformly from a short distance to a long distance.

In evaluating the performance of the proposed method, it was important to objectively compare to the existing methods. However, it was difficult to directly compare the reported performance of other works due to different types of cameras, and sensors, and different experimental conditions such as driving area, and weather conditions. Therefore, in addition to the evaluation based on the recognition distance, the recognition performance for objects with the similar pixel size as other works was also evaluated. Table 3 summarizes the characteristics of the pixel size of bounding boxes in the existing data set and the test data. The table indicates that the test data in this work were challenging data that included objects with large and small pixel sizes for traffic lights and arrow lights, even compared to existing public data sets.

The evaluations carried out in this work are summarize bellow:

- Analysis of the contribution to the recognition rate by using spatial prior information in the proposed method
- Comparison between the proposed method and a general object detection algorithm using DNN (YOLOv3 [28])
- Performance comparison of each candidate object detector (SURF, blob, AdaBoost) in the proposed method
- Comparison of processing time in recognition.

YOLOv3 was adopted as one of the state-of-the-art methods because it is a widely used DNN in object detection. The ML detector and the arrow detector (AdaBoost) of the proposed method and YOLOv3 were generated using the training data in Table 2. As described above, AdaBoost detectors were divided into two types (the TL detector and the arrow TL detector). The YOLOv3 model was generated as a model that recognizes six classes of TLs and arrow TLs. In the evaluation of the recognition rate, the data set was divided into intervals of 10 m, and precisions, recalls, and f-values for the data in each interval was used as an evaluation measure. The evaluation metrics were calculated by the following equations.

(18) $$\begin{array}{cc} Precision & =\frac{TP}{TP+FP} \end{array}$$

(19) $$\begin{array}{cc} Recall & =\frac{TP}{TP+FN} \end{array}$$

(20) $$\begin{array}{cc} F-\mathrm{value} & =\frac{2Recall\cdot Precision}{Recall+Precision}, \end{array}$$

where *TP* is the number of true-positives, *FP* is the number of false-positives, and *FN* is the number of false-negatives. The *TL* and the arrow *TL* were evaluated separately due to the difficulty in recognizing and the different number of data.

The relevant parameters of the proposed method were set as follows. $d_{T}=150$ m, $\theta_{T}=30$ deg, $k_{roi}=3.0,s_{s}=1.2$ m, $s_{l}=0.4$ m, $H_{min}=20000$, $k_{max}=0.5$, $k_{min}=2.5$, α=0.8. The computer used for the evaluation was a Windows 10 desktop PC, the CPU was Intel Xeon CPU E5-1620v4 (3.50 GHz), the memory was 16 GB, and the GPU was NVIDIA GeForce GTX TITAN X. The processing on the CPU was operated in a single thread.

### 4.2. Results

**Figure 13:**
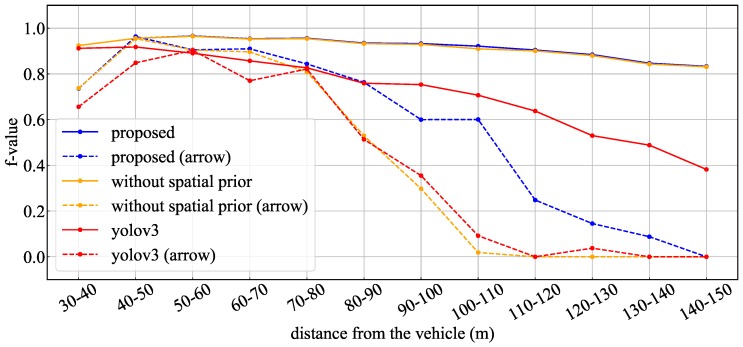
F values for whole data: with or without the prior information.

**Figure 14:**
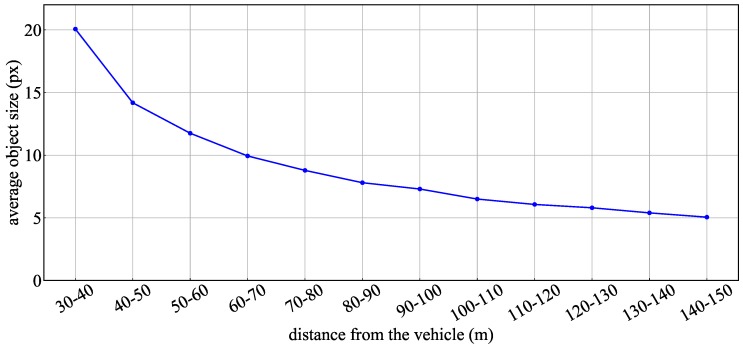
Average pixel size of the lighting area at different distances.

Figure 13 and Table 4 show the experimental results using the proposed method and YOLOv3 for the whole test data. Figure 13 indicates the recognition rate for each interval of the TLs and arrow TL, and Table 4 indicates the average of precision, recall and F-values obtained in each interval. In the TL recognition, the recognition rate of the proposed method was more than 90% even at approximately 100 m. Although the recognition rate decreased as the distance increased, it was confirmed that 80% or more could be maintained even 150 m away. Comparing the effects with and without spatial prior information, the precision, recall, and F-values were slightly improved by using spatial prior information. Therefore, a slight improvement of false-positive and false-negative detections was confirmed using spatial prior information. On the other hand, in YOLOv3, it was confirmed that a similar level of recognition rate was obtained at short distances of less than 40 m. However, the reduction of the recognition rate became larger as the distance increased compared to the proposed method. Comparing the recognition rates of the arrow TLs, it was confirmed that there was a large difference between the proposed method and other methods. Here, Figure 14 shows the average number of pixels of the lighting area in the TLs at each distance. In the object detection using machine learning, it was confirmed that the recognition rate was extremely low for arrow TLs smaller than 10 pixels. However, in the proposed method, it was found that the recognition rate at around 100 m could be greatly improved by suppressing the performance degradation. Therefore, it was shown that the proposed method, which uses the relative position between the TL and the arrow TL as prior information, can improve the recognition distance by 10–20m.

Next, we objectively evaluate the recognition results of this study against the performance reported in existing works. de Charatte reported a precision of 95.38% and a recall of 98.41% (37.4 ms per frame) as a recognition performance of LARA, a French dataset [13]. In addition, Chen reported a recognition rate of 95.75% (3 Hz per frame) for WPI, a USA dataset [14]. According to Table 3 and Figure 14, in our test data, the data within the range of 120 m (6 pixels or more) correspond to the difficulty of LARA, and the data within 60 m (11 pixels or more) correspond to the difficulty of WPI. Table 5 summarizes the recognition rates of the proposed method for each range of test data. The evaluation results for our test data indicates that the precision value was 97.1% for data within 60 m and 95.7% for data within 120 m. Although there were differences due to the different driving environment, it was shown that approximately the similar precision was obtained. In particular, it has been reported that the processing time of 3 Hz is required for the method [14] that can recognize both TLs and arrow TLs using the PCANet [15] which is a compact DNN model. On the other hand, in the proposed method, it was possible to simplify the arrow detector model by using prior information, and then the average processing time was 67 ms. Therefore, the proposed method achieved a recognition rate similar to SoAin a compact processing.

**Figure 15:**
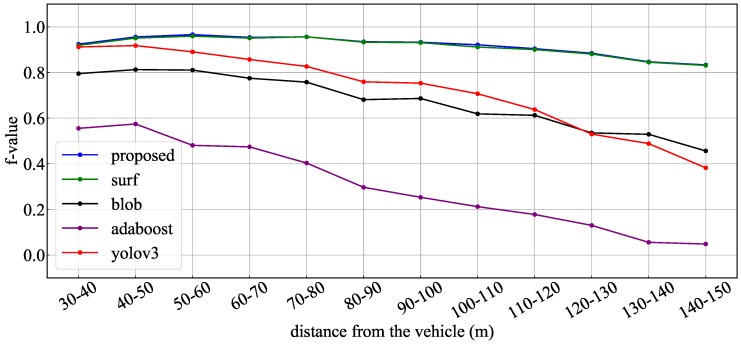
F values for whole data: different candidate extraction methods.

**Figure 16:**
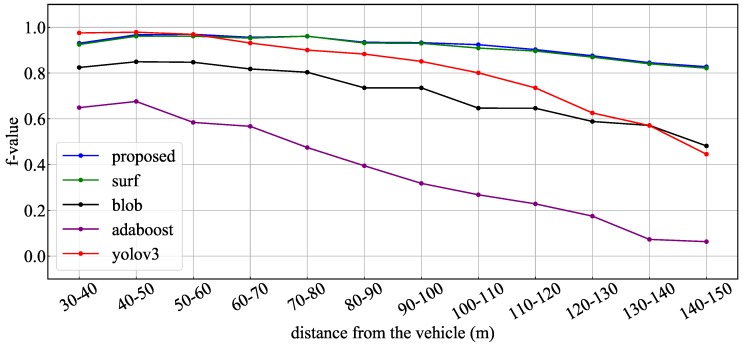
F values for daytime data: different candidate extraction methods.

**Figure 17:**
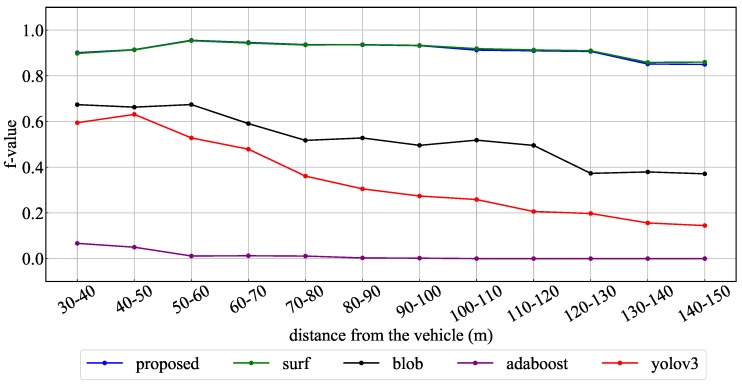
F values for dark data: different candidate extraction methods.

In order to detect candidate objects, the proposed method combined three kinds of methods: circular features by SURF, blob features by binarized images, and features of traffic signal shape by AdaBoost detector. To evaluate the contribution of each method, the obtained recognition rates were compared with the results obtained when each detection method was used alone. Figure 15, Figure 16 and Figure 17 show the obtained recognition rates for each detection method. These graphs indicate the recognition rate for all test data, the daytime scene data, and the dark scene data, respectively. Table 6 summarizes the average precision, recall, and F-value for the recognition result under each condition. From these results, it was confirmed that the method of circular extraction by SURF showed almost the same level of performance as the proposed method. Introducing the proposed method improved the average recognition rate by approximately 0.4%.

**Figure 18:**
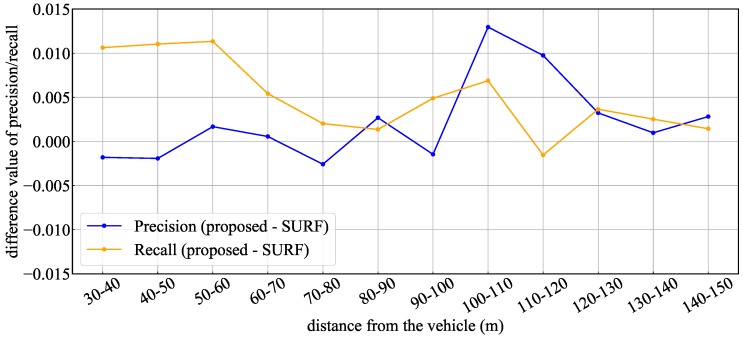
Difference value of precision/recall between the proposed method and sped up robust features (SURF).

Although the recognition rate of the proposed method and SURF were almost identical, a detailed analysis was performed to verify the superiority of the proposed method. Figure 18 shows a graph summarizing the different precision and recall values between both methods. In this figure, it can be confirmed that the proposed method is superior for short-ranges recall and long-ranges precision values. This means that false-negative rate at short-range and false-positive rate at long-range have been improved. Circular extraction by SURF enables appropriate detection by obtaining a feature image in which the lighting area is sufficiently enhanced. However, in the case of a lamp-type traffic light with weak brightness, the emphasis in the feature image may not be sufficient, and a false-negative detection occurs. In such a case, if the overall shape of the TL is visible, improvement of the false-negative detection is expected by recognizing the TL with the ML-detector. On the other hand, in the case of distant recognition, it is achieved that feature points of blobs as well as circular features of SURF are detected simultaneously. As a result, the recognition rate was improved because the false detection of the background and the true detection points could be differentiated for the obtained candidates. Thus, although the overall improvement rate is small, the introduction of the proposed method improved the recognition rate in specific situations and thus it proves to be a robust method.

**Figure 19:**
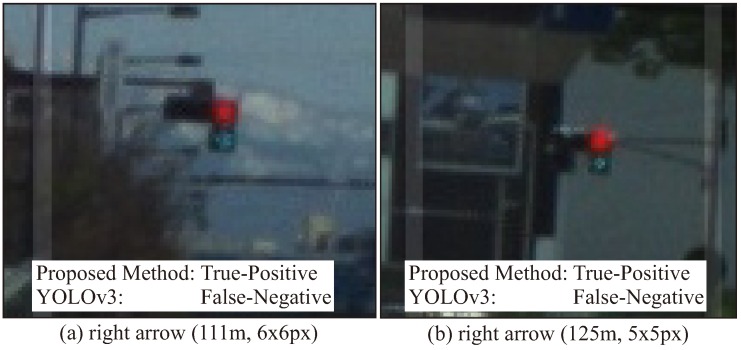
Typical improvement for far arrow TLs.

**Figure 20:**
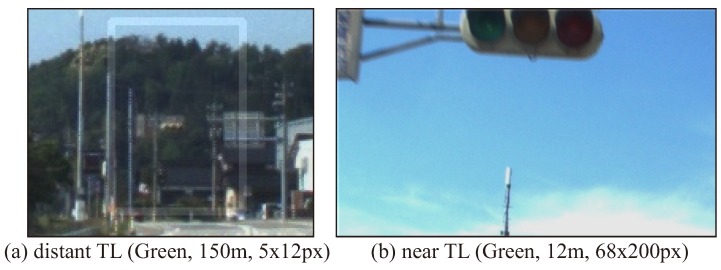
Typical false-negative situations due to low brightness of lamp-type TLs.

Figure 19 shows typical scenes of true-positives for small arrow detection. By utilizing prior information, the proposed method can detect small arrow TLs of 10 pixels or less. As shown in Figure 19, it was confirmed that an arrow of 5 pixels could be recognized. However, according to the evaluated results, there were difficult scenes where false-negatives occurred. Figure 20 shows typical false-negative images. On suburban roads, old ramp signals are installed. Such traffic lights have very low brightness, and in some cases there are images where it is difficult for humans to see the lighting color. In such a situation, even if the overall shape of the TL could be detected, it was false-negative because the lighting color was not bright enough.

Finally, the processing time for each method has been evaluated. Table 7 shows the average processing time and standard deviation of each method. In this experiment, the images have been captured at 7.5 Hz Then, if that could be processed within 133 ms, it would be a method that can be said to operate in real-time. According to Table 7, the real-time operation is possible because the average processing time of all methods was within 100 ms. However, while YOLOv3 required processing on the GPU, other methods, including the proposed method, could be operated with only the CPU. Therefore the proposed method was practically easier. Automated vehicles are required to process many recognition and decision-making processes in a limited computer environment. In this respect, usefulness was shown by analyzing the recognition performance and processing performance of the proposed method. According to Table 7, there were cases where the proposed method took about 100 ms instantaneously. There was no critical problem on the decision of approaching the intersection, because the moving amount of the vehicle during that time was about 1 or 2 m. The validity of the TL recognition during automated driving was evaluated in the next section.

### 4.3. Verification for Automated Driving

**Figure 21:**
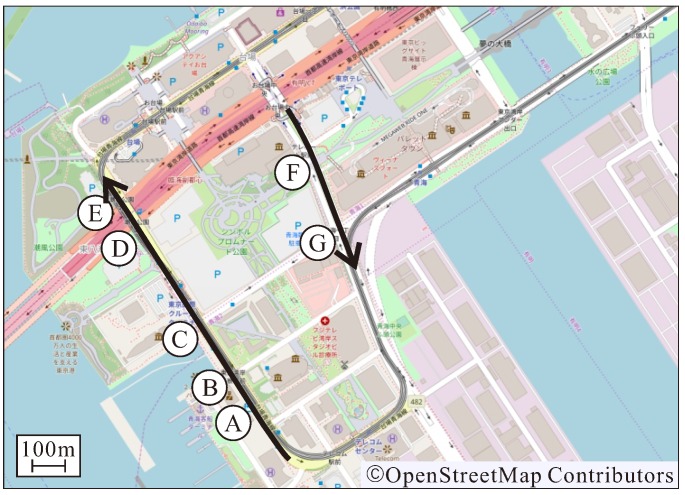
Driving routes for verification experiments.

We showed the superiority of the proposed method by evaluating a recognition distance for TLs and arrow TLs. Next, additional verification was carried out to determine if the proposed method has the required performance in actual automated driving. In the verification experiment, automated driving was performed in the Odaiba area of Tokyo as shown in Figure 21. In the automated driving, the role of the TL recognition is to determine the intersection approach following the traffic rules. If the signal state at the intersection is not properly recognized, the decision to enter the intersection will be incorrect and unnatural acceleration/deceleration will occur. In order to evaluate the effects of such a situation, it is necessary to investigate the stability of the TL recognition results and the vehicle acceleration when the automated vehicle is driving at the maximum velocity (the speed limit). Therefore, the transition of velocity and acceleration when passing through an intersection is verified while recognizing TLs.

Driving data were measured on the following two types of travel routes.

- Route passing through intersections A to E with only TLs in Figure 21
- Route passing through intersections F to G with TLs and arrow TLs in Figure 21.

**Figure 22:**
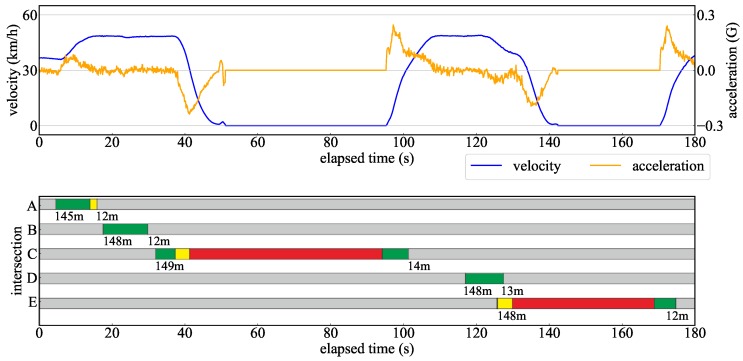
Verification results for automated driving from the intersection A to E.

**Figure 23:**
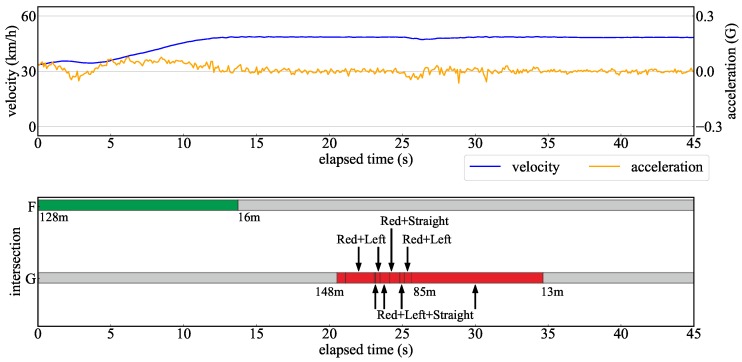
Verification results for automated driving from the intersection F to G.

In both routes, there was no preceding vehicle, because the automated vehicle should drive at the maximum velocity. Then it was verified whether the vehicle could drive smoothly while properly recognizing each TL. In this verification experiment, a camera different from Section 4.1 was used owing to a hardware issues. A camera with a resolution of 1920 × 1080 was installed in this experiment. It has a similar vertical field of view compared to the camera described in Section 4.1. Therefore, the recognition distance was extended by the ratio of the resolution ratio compared to the recognition distance in Section 4.2. Figure 22 and Figure 23 show the velocity and acceleration of each driving data and the TL state recognized at each intersection as verification results. The bar graph shows the TL status recognized at each time, the color of the graph is the light color, and the numerical value below it is the distance to the corresponding TL. According to Figure 22, it can be confirmed that the TL state was recognized immediately within 150 m of the recognition range. At the intersections C and E, the vehicle stopped at a red light. In this case, the vehicle stopped smoothly with moderate deceleration. On the other hand, Figure 23 shows a driving scene passing through an intersection while recognizing an arrow light in the straight direction. As a result of the evaluation in Section 4.2, the recognition performance of the arrow light was deteriorated for distant objects. Therefore, the recognition result at a point 100 m away at the intersection G was unstable. From the experimental results, it can be confirmed that the recognition result became stable at a point 85 m away. Even in such a situation, it was shown that unnecessary deceleration did not occur according to the transition of speed and acceleration. Thus, it was shown that the recognition result obtained by the proposed method had the necessary performance to drive smoothly at intersections in the urban areas.

In addition, in our group’s work, demonstration experiments of automated driving have been conducted in the area shown in Figure 21 since September 2019. In the half-year, there were no critical problems regarding the recognition distance and stability of the TL recognition. In such respect, the validity of the proposed method was qualitatively evaluated.

## 5. Conclusions

This work has proposed a TL recognition algorithm for urban automated driving. We prepared the challenging dataset that includes objects with large and small pixel sizes of objects for traffic lights and arrow lights. The proposed method can be processed in real time by the CPU, and our work verified that the proposed method can recognize TLs within 150 m with an F-value of 91.8%. This f-value is the recognition rate in one frame. When approaching an intersection from a distance of 150 m, recognition process of about 100 frames is performed, then the state of the intersection can be estimated with high confidence. The evaluations verify the following as the contributions of the work.

- Robust recognition was achieved by integrating multiple types of detection methods to recognize TLs including the small size of objects with a few pixels.
- Arrow TL recognition using prior information obtained from the HD map was proposed, and it was shown that small arrow object can be detected even if their size is smaller than 10 pixel.
- It was verified that the proposed method satisfies the necessary requirements for smooth deceleration of approximately 0.2 G at intersection approaches in urban automated driving.

In the arrow recognition by the proposed method, the arrangement pattern of the signal light and the arrow light given in Figure 2 was used as prior information to improve the recognition rate for distant objects. The map information corresponding to such prior information can be used practically, because it is included as static information of the HD map that has already been studied [31]. On the other hand, the evaluation in this experiment showed a recognition rate of more than 90%, but there were cases where recognition became difficult. In addition to Figure 20, there are cases where it is relatively difficult to determine the lighting color of the lamp due to the influence of the surrounding brightness. For example, in the case of receiving the sunlight from behind the vehicle, all lamps may appear to be lit. Moreover, there are cases where yellow and red lighting colors can be visually recognized to the same color in the images. These cases will be a false-positive detection. In addition to the issues of software based recognition algorithms, but also the performance limit of hardware needs to be discussed from a practical point of view. As described in [32], there are situations in which it is difficult to view the TLs in the image in severe sunshine as a hardware performance limit. It is desirable to develop a practical recognition methods while discussing such software and hardware issues. 

## Figures and Tables

**Table 1:** Summary of relative studies for traffic light recognition.

Paper	Method	Object	Resolution	Time	Distance/Min. Pixel	Accuracy
[13], 2009	Blob candidate; Adaptive Template Matcher	TL	640 × 480	37.4 ms (CPU)	6 px	95.38% (Prec.) 98.41% (Recall)
[9], 2011	Blob candidate; Mapping	TL; Arrow	2040 × 1080	4 Hz (CPU)	200 m	99% (Prec.) 62% (Recall)
[10], 2011	Probabilistic Template Matching; Mapping	TL	1.3 megapixel	15 Hz (CPU)	140 m	91.7%
[11], 2014	Blob candidate; CNN; Prior Map	TL	—	300 ms (CPU)	100 m	99.4%
[14], 2016	Blob candidate; PCANet [15]; Multi-Object Tracking	TL; Arrow	1920 × 1080	3 Hz (CPU)	13 px	95.7% (Prec.) 95.7% (Recall)
[16], 2019	SSD [17]; Prior Map	TL	1368 × 1096	17.9 ms (GPU)	150 m	86.9%
[18], 2017	YOLO [19]; DNN Classifier; Tracking	TL; Arrow	1280 × 720	15 Hz (GPU)	4 px	—
Ours	Circle, Blob & Shape candidate; AdaBoost; Prior Map	TL; Arrow	1280 × 960	64 ms (CPU)	150 m 2 px	91.8% (TL) 56.7% (Arrow)

**Table 2:** Experimental conditions: number of data.

Train/Test	Scene	Green	Yellow	Red	Left	Straight	Right
Train	Daytime	10,211	422	5277	129	293	194
Test	Daytime	30,506	1867	17,721	662	219	890
Test	Dark	9206	646	5005	49	153	247
Test	Total	39,712	2513	22,726	711	372	1,137

**Table 3:** Bounding box pixel size in test dataset and other open dataset.

Dataset	Classes	Num. of Objects	W/H	Minimum	Average	Median	Maximum
Our test data	6	67,171	Width	2.82	34.75	26.72	213.02
(Day & Night)	(3 lights & 3 arrows)		Height	2.22	15.22	11.94	174.98
LARA [13]	4	9,168	Width	6.00	11.45	11.00	37.00
(Only Datytime)	(3 lights & ambiguous)		Height	14.00	27.24	28.00	93.00
WPI [14]	6	4,207	Width	13.00	28.03	28.00	47.00
(Only Datytime)	(2 lights & 4 arrows)		Height	13.00	27.30	28.00	48.00
Bosch [18]	4	13,493	Width	1.88	9.43	8.50	48.38
(Only Datytime)	(3 lights & off)		Height	3.25	26.75	24.50	104.50

**Table 4:** Experimental results with or without the prior information.

Object	Method	Mean Precision	Mean Recall	Mean F-Value
traffic light	proposed	0.938	0.899	**0.918**
	without spatial prior	0.937	0.894	0.915
	YOLOv3	0.972	0.598	0.722
arrow light	proposed	0.771	0.517	**0.567**
	without spatial prior	0.542	0.386	0.429
	YOLOv3	0.611	0.352	0.417

**Table 5:** Experimental results of the proposed method in different distance ranges.

Object	Distance		Mean Precision	Mean Recall	Mean F-Value
traffic light & arrow light	30–150 m	(ave. pixel size > 5.0)	0.935	0.897	0.910
traffic light & arrow light	30–120 m	(ave. pixel size > 6.0)	0.957	0.907	0.932
traffic light & arrow light	30–60 m	(ave. pixel size > 11.0)	0.971	0.920	0.945

**Table 6:** Experimental results: different candidate extraction methods.

Scene	Method	Mean Precision	Mean Recall	Mean F-Value
Whole	proposed	0.938	0.899	**0.918**
	SURF	0.936	0.894	0.914
	blob	0.917	0.536	0.672
	AdaBoost	0.990	0.194	0.305
	YOLOv3	0.972	0.598	0.722
Daytime	proposed	0.930	0.902	**0.915**
	SURF	0.928	0.899	0.913
	blob	0.921	0.587	0.712
	AdaBoost	0.990	0.250	0.372
	YOLOv3	0.986	0.710	0.806
Dark	proposed	0.959	0.871	0.913
	SURF	0.958	0.875	**0.915**
	blob	0.902	0.372	0.523
	AdaBoost	0.583	0.007	0.013
	YOLOv3	0.840	0.226	0.345

**Table 7:** Experimental results: computational time.

Method	CPU/GPU	Average (ms)	Standard Deviation (ms)
proposed	CPU	64.278	19.374
SURF	CPU	61.036	16.890
blob	CPU	45.806	10.267
AdaBoost	CPU	71.663	22.322
YOLOv3	CPU/GPU	93.945	29.693
